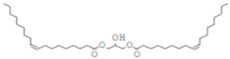# Preparation of KI/Hydroxyapatite Catalyst from Phosphate Rocks and Its Application for Improvement of Biodiesel Production

**DOI:** 10.3390/molecules25112565

**Published:** 2020-05-31

**Authors:** Widayat Widayat, Hadiyanto Hadiyanto, Permadi Wisnu Aji Wardani, Ummi Az Zuhra, Jedy Prameswari

**Affiliations:** 1 Chemical Engineering Department, Faculty of Engineering, Diponegoro University, Semarang 50271, Indonesia; wisnupandhef@students.undip.ac.id (P.W.A.W.); ummiazzuhra@students.undip.ac.id (U.A.Z.); 2 Center of Biomass and Renewable Energy, Diponegoro University, Semarang 50271, Indonesia; 3 Advanced Materials Research Laboratory, Diponegoro University, Semarang 50271, Indonesia

**Keywords:** biodiesel, HAP, impregnation KI/KIO_3_, simultaneous reaction, waste cooking oil

## Abstract

The main aim of this work was to investigate the suitability of a KI/KIO_3_ impregnated hydroxyapatite (HAP) catalyst derived from natural phosphate rocks for biodiesel production. This study evaluated the effect of impregnation concentrations (1–6% *w*/*w*) on the catalyst performance in biodiesel production. The biodiesel was produced from waste cooking oil (WCO) under simultaneous esterification-transesterification reactions at 60 °C for 6 h. The results showed that the biodiesel yield increased by increasing impregnation concentration and the maximum yield (91.787%) was achieved at an impregnation concentration of 5% *w*/*w*. The KI/HAP catalyst showed better performance (91.78% biodiesel yield, 59.1% FAME yield and surface area of 13.513 m^2^/g) as compared to the KIO_3_/HAP catalyst (90.07% biodiesel yield, 55.0% FAME yield and surface area of 10.651 m^2^/g).

## 1. Introduction

Increasing demand for fossil fuel has led to extensive energy exploration. Biodiesel or fatty acids methyl esters (FAME) is a vegetable-derived fuel and considered as an alternative for conventional diesel fuel. Several advantages of biodiesel include the high flash point, biodegradability and lower gas emission [1]. An oil source which contains high free fatty acids (FFAs) such as waste cooking oil (WCO) is now being used for biodiesel synthesis, as they are less expensive than refined oil, and it offers significant advantages i.e., reduction in environmental impacts. Biodiesel from waste cooking oil has been successfully derived with simultaneous esterification and transesterification process using a homogenous catalyst [2]. Conventionally, the biodiesel synthesis uses homogenous catalyst either acid or base catalysts. The homogenous acid catalysts for esterification are H_2_SO_4_, HCl, H_3_PO_4_, HNO_3_ and the base catalysts are KOH and NaOH. The drawbacks of these homogenous catalysts are long reaction times (3–4 h) and they require complex separation of final products and neutralization process [3,4]. Although liquid acid catalysts likely give higher biodiesel yield, the corrosiveness and slower reaction are the main disadvantages as compared to the base catalyst [4,5]. As a consequence of these drawbacks, many studies are being developed to investigate heterogeneous catalysts because of their non-corrosive properties, large surface area and easiness of separation from biodiesel [4,6]. The common heterogeneous catalysts are immobilized lipase, calcium and magnesium oxides, as well as ion exchange resins [4,5,7]. However, the heterogeneous catalysts are mostly used only on the transesterification reaction and therefore, it is required to investigate the application of these catalyst on simultaneous reactions of esterification and transesterification for efficient and economical considerations [8].

Hydroxyapatite Ca_10_(PO4)_6_(OH)_2_ (HAP) is a low-cost mineral catalyst in biodiesel synthesis which could be derived from eggshells, prawn shells, animal bones, mollusk shells and phosphate rocks. Phosphate is an element found in frozen rock sediments containing apatite mineral, which is abundant in nature. It is approximated that the world has a natural phosphate rocks deposit of 87,810 million tons, with the largest deposits in North Africa (41.453%), followed by West Africa (20.840%) and North America (14.805%). Indonesia has a deposit of 18.961 million tons of phosphate rocks, accounting for 0.774% of the total deposit in the world, with a P_2_O_5_ content ranging from 6.25% to 49.3% [9]. In Sukolilo, Pati, Central Java, Indonesia, there is a deposit of 85,000 tons of phosphate rocks with a 25–35% P_2_O_5_ content. The utilization of phosphate rocks in Indonesia is still limited to phosphoric acid and phosphate fertilizer production [9], therefore ensuring the sustainability of phosphate rocks as raw material. Recent studies have found HAP to be a potential catalyst for biodiesel production due to its advantage in ion-exchange, adsorption capacity, non-toxic nature and thermal stability [10]. Moreover, the strong acidic active site of HAP will greatly benefit the esterification process in the simultaneous reaction. Essemlali et al. [11] conducted a piece of research on Na/HAP catalyst synthesis by evaluating the effect of mass ratio between NaNO_3_ and calcination temperature and obtained biodiesel yield above 90%. Chen et al. [12] also conducted a piece of research on synthesizing a K/HAP catalyst from animal bones and obtained a biodiesel yield above 90%.

In this study, KI and KIO_3_ were used with HAP (as support) for biodiesel catalysis, due to the fact that potassium was relatively more effective [12]. Moreover, KI and KIO_3_ have shown high catalytic activity in transesterification reaction due to its strong basic active sites, as evidenced by Islam et al. [13] who obtained a 98% biodiesel yield using a KI/*γ*-Al_2_O_3_ catalyst. Malani et al. [14] used a KI/ZnO catalyst resulting in 92.35% biodiesel yield, Tantirungrotechai et al. [15] used a KI/Mg-Al catalyst resulting in an above 90% biodiesel yield and Jairam et al. [16] used a KI/oyster shell catalyst resulting in 85% biodiesel yield. Therefore, KI and KIO_3_ supported by HAP have shown their potencies as catalyst for simultaneous reaction heterogeneous catalyst.

This research aimed to (i) evaluate the effect of varying KI and KIO_3_ impregnation concentration to the HAP catalyst, (ii) analyze the characteristics of the catalysts with XRD, SEM, BET and FTIR analyses, and (iii) apply the catalyst to biodiesel production through a one step simultaneous esterification-transesterification process.

## 2. Materials and Methods

### 2.1. Materials

Natural phosphate rocks used in this research were taken from Sukolilo, Pati Regency, Central Java, Indonesia. Waste cooking oil (WCO) was obtained from several restaurants in Tembalang, Semarang, Indonesia. Other materials used were distilled water, HNO_3_ (65%, Merck, Darmstadt, Germany), NH_4_OH (25%, Merck, Darmstadt, Germany), KIO_3_ (99.8%, Merck, Darmstadt, Germany), KI (99.5%, Merck, Darmstadt, Germany) and methanol (99.9%, Merck, Darmstadt, Germany) which were bought from the Multi Kimia Raya store in Semarang, Indonesia. All chemical reagents used were analytical grade.

### 2.2. HAP Catalyst Synthesis

The method to synthesize the HAP catalyst followed Rivera et al. [17] with several modifications. In Rivera et al. [17], the eggshells were first heated at 2 stages, first at 450 °C for 2 h at a rate of 5 °C/min and second at 900 °C for 2 h at a rate of 0.5 °C/min. After it was added to a solution, it was heated again to 1050 °C for 3 h at a rate of 10 °C/min. While in this research, 50 g of phosphate rocks were ground and sieved to the distribution size of 100–400 μm. Then it was dissolved slowly in 500 mL of acid solution (HNO_3_ 1 M, pH 2) with high-speed stirring to maintain the Ca^2+^ and H_3_PO^4−^ ions. The solution was filtered and NH_4_OH was added to the filtrate until the pH reached 10 and left for 24 h until a white precipitate was formed. The precipitate was filtered and washed with distilled water until the pH of the filtrate was equal to the distilled water pH, then dried at 110 °C. Calcination followed at 1050 °C for 4 h to produce the HAP catalyst. The next step was impregnation by dissolving KI and KIO_3_ solution in 50 mL of distilled water on concentration 1–6% (*w*/*w*). About 20 g of HAP was then mixed into the KI and KIO_3_ solution, stirred and heated for 2 h at 90 °C. The solution was filtered with filter paper and the residue was dried.

### 2.3. Catalyst Characterization

The catalyst characterized was the non-impregnated catalyst and the catalyst impregnated with KI and KIO_3_. Catalyst characterization by XRD (X-ray diffraction, Bruker AXS D8 Diffractometer, Billerica, MA, USA) aimed to determine the crystallinity of the catalyst through the peaks at 2*θ*. While SEM (scanning electron microscopy, (JSM-5410, JEOL, Tokyo, Japan) analysis was carried out to determine the surface morphology and pore distribution on the hydroxyapatite [12]. For other characterizations, FTIR (Perkin Elmer, Waltham, MA, USA) and BET (Quantachrome NOVA 1000 High- Speed Gas Sorption Analyser, Boynton Beach, FL, USA) were used to determine the functional group and pore size of the catalyst surface.

### 2.4. Biodiesel Synthesis

Biodiesel synthesis was carried out to test the performance of the catalyst. The method used was a one step simultaneous esterification and transesterification reaction in a batch reactor according to research conducted by Konwar et al. [2] with several modifications. Methanol and WCO with a mole ratio of 10:1 were prepared with the catalyst (1% *w*/*w* of WCO). The mixture was fed into a three-necked flask and heated until 60 °C with stirring at 800 rpm. The reaction will be terminated after 6 h. To separate the product and the catalyst, the mixture was filtered with filter paper while glycerol was also separated in a separation funnel. Distillation was used to separate methanol from biodiesel products. The solid catalyst was then washed with n-hexane for reuse.

### 2.5. Biodiesel Characterization

The product was analyzed with GC-MS (gas chromatography mass spectrometry, Agilent 7890A, Wilmington, DE, USA) to determine the composition of FAME in the product. Biodiesel yield can be calculated using Equation (1):

Yield = (Biodiesel weight)/(WCO weight) × 100%(1)

Other biodiesel properties were also evaluated such as density, kinematic viscosity and FFA.

## 3. Result and Discussion

### 3.1. Functional Group Analysis

**Figure 1:**
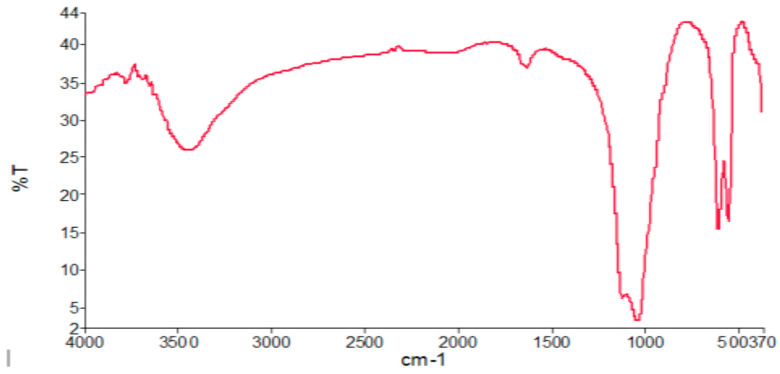
FTIR analysis result of HAP.

FTIR analysis (Figure 1) showed the functional groups in the HAP catalyst. The HAP functional group spectrum has a distribution of several points, which could be seen from the peak distributions alongside the wavenumber. Based on Figure 1, transmittance at 476–1035.75 cm^−1^ indicated there was a stretching and bending on the phosphate group (PO4^3−^), transmittance at 1421.2–1637.78 cm^−1^ showed that the functional group was carbonate (CO^3−^) and the absorbance at 3448–3460.79 cm^−1^ identified a hydroxyl group (OH^−^). These results show that the synthesized HAP had a high purity, which was in accordance with the research results of Yielmaz and Yilmaz [18] and Darwis et al. [19], although there was still a carbonate functional group present at a very small amount (trace element).

### 3.2. XRD Analysis

**Figure 2:**
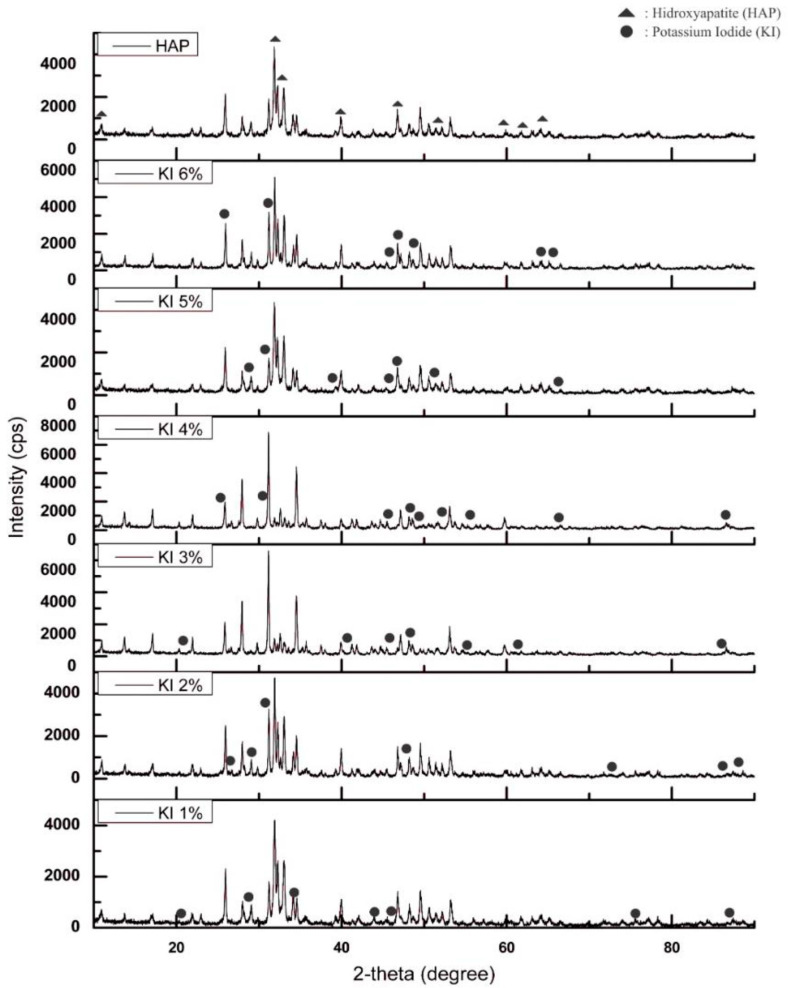
XRD pattern of HAP and KI/HAP catalyst.

The XRD pattern was illustrated on the diffractogram profile (curve with peaks), with diffraction angle 2*θ* as the *x*-axis and diffraction intensity as the *y*-axis. Figure 2 shows the analysis of the diffractogram pattern of HAP and KI impregnated HAP catalysts. On the XRD pattern of the HAP catalyst without impregnation, the highest intensity peak identified belonged to hydroxyapatite, which was 31.854° (Ca_5_P_3_HO_13_) and 32.970° (Ca_5_P_3_HO_13_), with a hexagonal crystal system, matching with the COD 9,002,213 data. Said HAP catalyst had lattice parameters of a = 9.4081 Å and c = 6.8887 Å and also a density of 3.159 g/cm^3^. The highest peak on 1% KI impregnated was identified at 2*θ* = 31.9° (Ca_5_P_3_HO_13_); 2% KI impregnation at 2*θ* = 31.9° (Ca_5_P_3_HO_13_); 3% KI impregnation at 2*θ* = 31.157° (Ca_10.115_P_7_Mg_0.386_O_28_); 4% KI impregnation at 2*θ* = 31.16° (Ca_10.115_P_7_Mg_0.385_O_28_); 5% KI impregnation at 2*θ* = 32.99° (Ca_5_P_3_HO_13_); and 6% KI impregnation at 2*θ* = 31.91° (Ca_5_P_3_HO_13_). In general, the highest peak of each KI impregnated sample was identified as hydroxyapatite, except for the 3% and 4% impregnation variables. However, the hydroxyapatite pattern was still present on the variables’ peaks.

**Figure 3:**
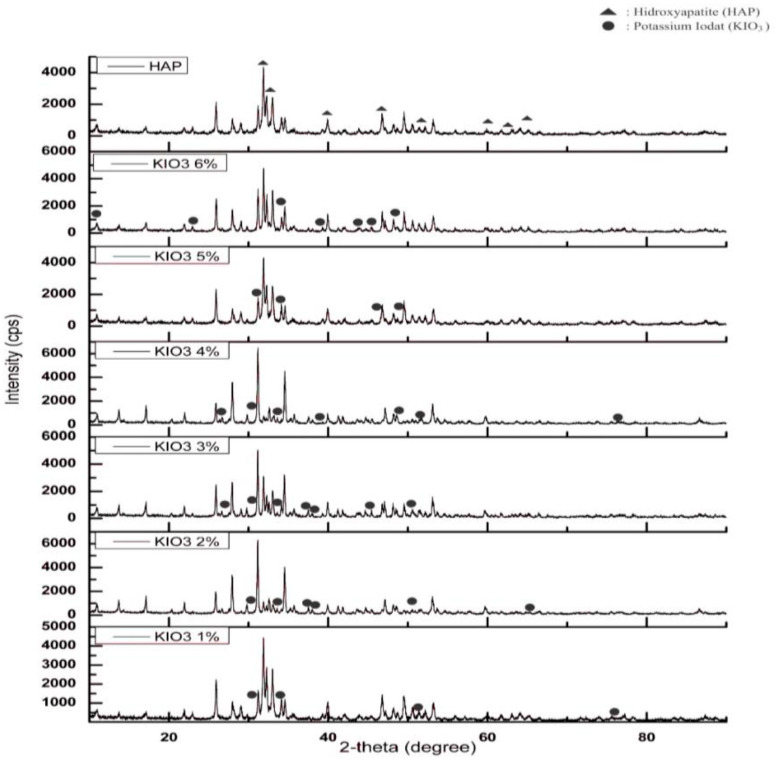
XRD pattern of HAP and KIO_3_/HAP catalyst.

Figure 3 shows the XRD pattern of the HAP catalyst which was impregnated with KIO_3_. On 1% and 5% impregnation, the highest peak was identified at 2*θ* = 31.890° which belonged to HAP, and the diffractogram pattern matched the hydroxyapatite pattern (COD 9002213). KIO_3_ impregnation at 6% resulted in the pattern and highest peak at 2*θ* = 31.830° which matched the synthetic hydroxyapatite (ICDD No. 01-076-0694). This was different compared to the impregnation of KIO_3_ on 2%, 3% and 4% where the highest peaks identified were at 31.149° (Ca_10.115_P_7_Mg_0.385_O_28_), 31.153° (Ca_10.115_P_7_Mg_0.385_) and 31.175° (Ca_10.115_P_7_Mg_0.385_) respectively. It could be due to the fact that during the calcination process, Mg was not eliminated and the hydroxyapatite crystal-forming reaction was not optimal. Hence, the functional group bonded to the apatite mineral was not the hydroxyl group, but Mg. Generally, for all the catalyst samples, KIO_3_ was shown as the peaks at 2*θ* = 34.530–34.550° and 2*θ* = 37.480–34.560°. The number of peaks and the intensity of KIO_3_ increased as the % *w*/*w* of impregnation increased [20].

### 3.3. Catalyst Surface Area and Pore Volume Analysis

Characterization of the surface area, catalyst pore volume and catalyst size were carried out with BET (Brunauer–Emmett–Teller) analysis. This analysis was based on the adsorption and desorption of nitrogen gas (N_2_) on a porous solid (wide pore range) at a normal boiling point. The BET analysis results are shown in Table 1.

According to IUPAC, the pore size of a porous solid could be categorized as micropore (d < 20 Å), mesopore (20 < dp < 500 Å) and macropore (dp > 500 Å). Based on Table 1, the HAP, KI/HAP (5%) and KIO_3_/HAP (6%) catalysts from this research had a pore radius of 16.330 Å, 7.38 Å and 6.83 Å respectively, and therefore fell into the micropore category. The surface area and pore volume from pure HAP (before impregnation) were 21.631 m^2^/g and 0.0330 cc/g. After it was impregnated with 5% KI and 6% KIO_3_, there was a significant decrease in surface area and pore volume, to 10.651 m^2^/g and 0.0065 cc/g. The decrease of surface area and pore volume can be tied to the changes on the surface of HAP which was covered by K compound. Furthermore, the effect of high-temperature sintering of HAP also led to catalyst particle agglomeration [11]. The obtained surface areas of the HAP, KI/HAP and KIO_3_/HAP catalysts were lower compared to the commercial HAP catalyst which typically had a surface area of >100 m^2^/g. This was attributed to the fact that wet impregnation was used as the method for catalyst synthesis, which resulted in the particles inevitably agglomerating hence yielding low a surface area, around 20–60 m^2^/g [21]. These findings were also reflected in a study of the HAP/K_2_CO_3_ catalyst conducted by Chen et al. [12], where the surface area of the catalysts was 4.02–30.74 m^2^/g with a decreasing trend along with rising sintering temperature and K_2_CO_3_ loading as it was covered with K compound.

### 3.4. Catalyst Surface Morphology Analysis

**Figure 4:**
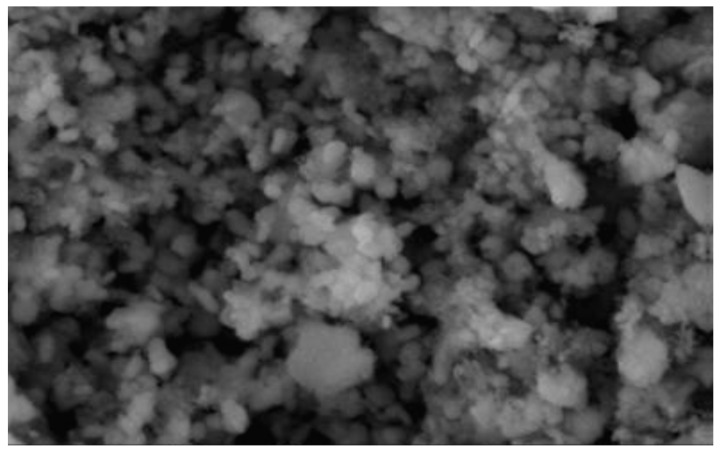
SEM result of the HAP catalyst.

The catalyst surface morphology structure was analyzed using SEM (scanning electron microscopy) with a 7500× magnification. The SEM observation results of the hydroxyapatite catalyst can be seen in Figure 4.

The SEM analysis based on Figure 4 showed that the hydroxyapatite catalyst from this research had a crystalline structure and irregular glob form, similar to the one obtained by Kmieniak et al. [22], however with a better particle dispersion. The catalyst particle was not clearly shown to be hexagonal shaped, similar to the XRD results due to the agglomerate formation on some particles. High-temperature calcination will give the sintering effect on HAP which could lead to agglomeration of catalyst particles [11]. Gupta et al. [23] also explained that the sintering phenomena could result in particles to be more circular shaped and has a smoother surface.

**Figure 5:**
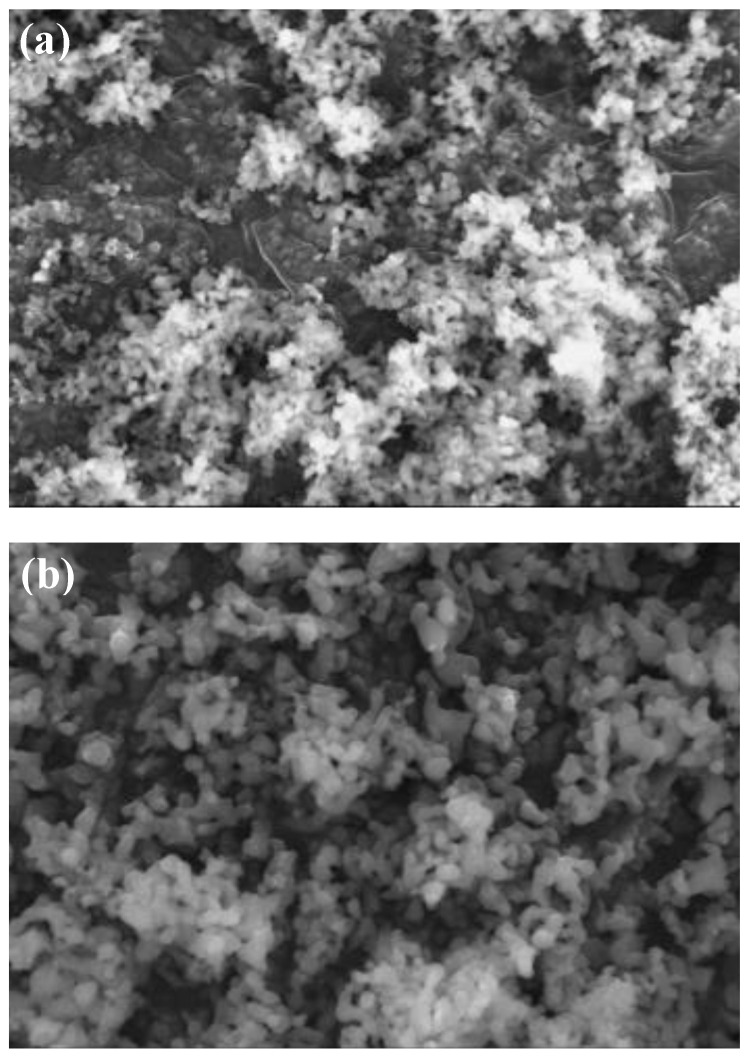
SEM result of impregnation.

The morphology of the catalyst surface which was impregnated with KI and KIO_3_ is shown in Figure 5 with a 10,000× magnification. The presence of white dispersed glob indicates that KI and KIO_3_ had been impregnated. In Figure 5a, the KI particle formed an agglomerate that covered most of the catalyst’s pore surface, while on Figure 5b the KIO_3_ particle formed a thin layer which was distributed evenly on the catalyst surface. Compared with the hydroxyapatite SEM results in Figure 4a, HAP which has been impregnated did not give a significant morphological difference. This was due to the fact that the impregnation concentration variance used was only 1–6% *w*/*w*. HAP which was impregnated with KI and KIO_3_ aimed to produce a basic property for the catalyst whilst maintaining a large surface area. The BET analysis results showed there was a decrease in HAP surface area after impregnation, but active surface for the catalysis reaction to happen was still present (sufficient).

### 3.5. WCO Characterization

The chemical composition of WCO was analyzed using GCMS, which is shown in Table 2. Other characteristics including density, viscosity and free fatty acid content were 0.944 g/cm^3^, 30.182 cSt and 4.489% respectively. Waste cooking oil used in this research was mainly composed of hexadecenoic acid (93.65%) which was palmitic acid. If compared to the research conducted by Abidin et al. [24], the fatty acid component of WCO was simpler. The difference between the composition of fatty acids contained in the WCO depended on the source of the oil, usage frequency and type of food fried in the oil as those factors will affect the chemical bonds and/or structure present in the oil [25].

### 3.6. Heterogeneous Catalyst Reaction Mechanism

**Figure 6:**
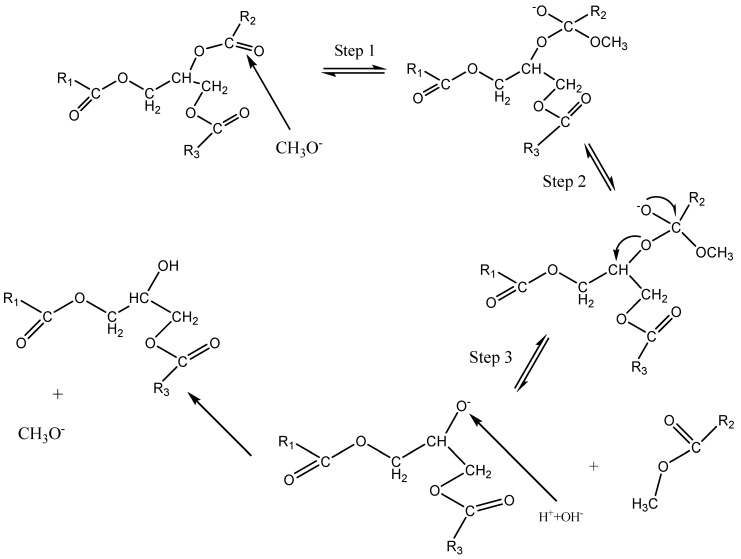
Biodiesel synthesis reaction mechanism.

HAP will act as an acid-base catalyst on a Ca/P ratio of 1.500–1.670. A low Ca/P ratio will decrease the basic property from HAP [26]. The acid active site of HAP will act on the esterification reaction and the basic active site on the transesterification reaction. The transesterification reaction started with the formation of OH^−^ or RO^−^ as an active element. Methanol will be absorbed on the surface of the catalyst and then the ion-exchange process will happen. Next, an active catalytic RO- element is formed. The active element formed will attack the carbonyl function on the first triglyceride chain to form an intermediate. Next, there will be re-arrangement of the chain due to the break-down of the oxygen chain and the addition of proton H^+^ to form FAME and diglyceride. The pathway would be repeated two times to form biodiesel and glycerol [27] (Figure 6).

Based on the chemical composition of the product on this research, the transesterification reaction which happened will not follow the general reaction pathway which was shown in Figure 6. This assumption was based on the GCMS analysis result which showed that a hexadecenoic acid, 2 hydroxy, 1,3 propanedixyl ester element was formed. The basic active site of CH_3_O^−^ directly attacked the second palmitic chain of the triglyceride. This caused the hydroxyl functional group (OH^−^) which formed the glycerol structure to only replace one ester molecule, hence the reaction will stop at this step. As a consequence, the yield produced had a high viscosity as glycerol was not perfectly formed and separated from the biodiesel.

### 3.7. Effect of Catalyst Type to Yield

**Figure 7:**
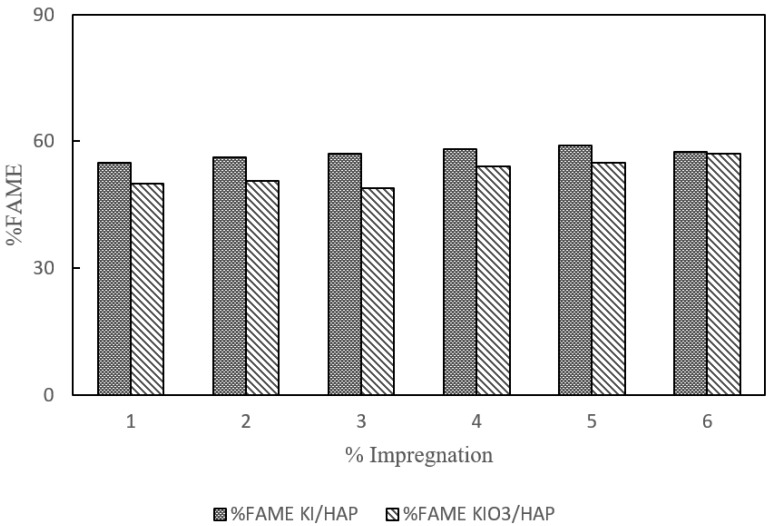
Effect of catalyst type on % FAME of biodiesel.

**Figure 8:**
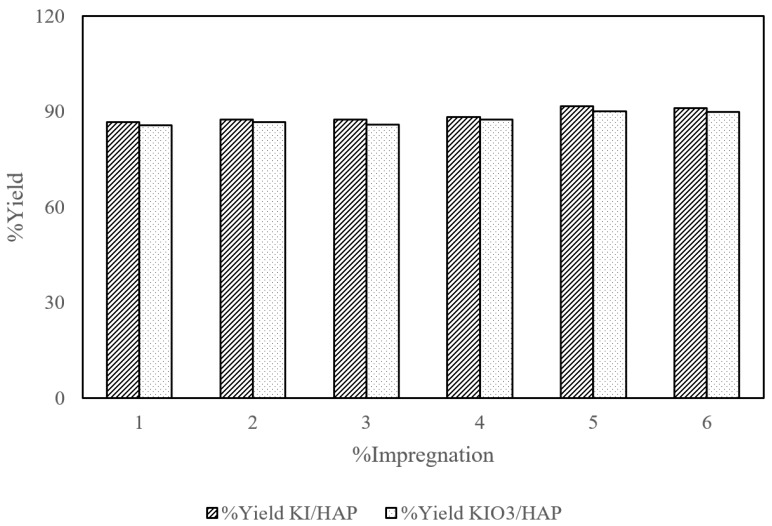
Effect of catalyst type to % yield of biodiesel.

The performance of each catalyst was evaluated from the yield of biodiesel produced and FAME content which was analyzed using GCMS for an impregnation concentration of 1% to 6% *w*/*w*. Figure 7 and Figure 8 show the effect of catalyst type towards % FAME and yield produced respectively. For the KI/HAP catalyst, the yield and % FAME showed an increasing trend up to 5% concentration; however, it decreased when the impregnation concentration was increased to 6%. While on the KIO_3_/HAP catalyst, the yield and % FAME tend to increase. The highest % FAME obtained from the KI/HAP catalysts and KIO_3_/HAP catalysts were 59.100% and 57.130% respectively. Both graphs are represented through a polynomial equation by following the fluctuating pattern change of yield and % FAME value on the catalyst impregnation concentration. The approach with polynomial regression was done to lessen the risks of determining an inaccurate target value caused by irregular data distribution. The fluctuation of yield and % FAME value on the product was caused by the evaporation of liquid phase KI/KIO_3_ when the impregnation process was carried out at a 90 °C temperature for 2 h. The evaporation which took place was due to the fact that there was too little addition of KI/KIO_3_. The evaporation phenomena which happened will affect the produced KI/KIO_3_ composition.

KI/HAP resulted in the highest yield due to the fact that KI was basic in nature and had high catalytic activity. Research conducted by Razak et al. [28] showed the same results on the catalyst which was supported by alumina and KI. The decrease which happened on the catalyst with KI impregnation of 6% may have been caused by a reaction between KI and free fatty acid (FFA). A K^+^ ion will react with the OH^−^ to form a basic substance that will inhibit the catalytic reaction on transesterification. As a consequence, there will be a saponification reaction in the product, resulting in a decrease in the product yield. Although both yield and FAME results have similar trends between the KI impregnated and KIO_3_ impregnated catalyst, these were also mirrored in a study by Widayat et al. [29] who synthesized KI/KIO_3_ impregnated zeolite H (1–5% *w*/*w*) and got similar biodiesel conversion trends between KI and KIO_3_ impregnated zeolite (80–87.91%). Other studies including biodiesel production with Al/KI and Al/CaO/KI catalysts have also shown similar trends between 80–90% yield with 1–3.5% KI loading [28] and biodiesel production with a KI/*γ*-Al_2_O_3_ catalyst which yields similar trends on % FAME yield with 0.15–0.33% *w*/*w* KI loading [13]. These similar trends were due to the fact that KI can readily dissolve in methanol, hence giving a highly basic nature leading to high adsorptive sites for alcohol transesterification that gave similar yield and % FAME trends.

### 3.8. Biodiesel Product Characterization

The biodiesel produced in this research was characterized by three test parameters: density, viscosity and FFA content. These parameters served as a comparison standard to the Indonesian National Standard (SNI 7182-2015) and European Standard (EN 14214:2008) of biodiesel. Based on Table 3 and Table 4 it can be seen that with increasing the catalyst impregnation percentage (KI or KIO_3_), the density and viscosity of the products decreased. The decrease of density was caused by triglyceride on the waste cooking oil being broken down to form methyl ester and glycerol. The products formed had a density range of 0.9032–0.9005 g/cm^3^ and viscosity of 28.080–20.790 cSt at room temperature. Waste cooking oil used as a raw material has an FFA content of 4.900%, while the FFA content of the product was in the range of 1.726–1.876% for KI impregnated and 1.759–1.815% for KIO_3_ impregnated. The FFA content of the product decreased on each increasing impregnation value for KI and KIO_3_.

According to the Indonesian standard, biodiesel has a density range of 0.850–0.900 g/cm^3^ and viscosity of 2.860 cSt at 40 °C, while the European standard stated biodiesel the density and viscosity values to be in the range of 0.860–0.900 g/cm^3^ and 3.5–5.0 cSt at 40 °C respectively. Hence the quality of products for variables 1–4% KI and 1–5% KIO_3_ produced did not meet either the Indonesian or European required standard. This phenomenon was caused by the first chain (R1) and the third chain (R3) on the triglyceride yet to be broken down, hence less glycerol and methyl ester produced. In Figure 6 it could be seen that methanol only attacked the second triglyceride chain (R2), therefore forming a substance with a high molecular weight, such as 1,3-propanedyl ester. The density value was directly proportional to the product mass, thus increasing the product mass will yield higher density over the same volume. The high viscosity of the product was due to the imperfectly broken-down triglyceride chain which led to the formation of glycerol along with FAME. As the viscosity of glycerol was higher than that of FAME, the presence of glycerol would increase viscosity.

### 3.9. Biodiesel Product Chemical Composition

Based on the chromatogram from the GCMS analysis, the biodiesel synthesized consisted of the components in Table 5. The methyl ester was formed from hexadecanoic acid, 9-octadecenoic acid and octadecanoic acid. Those results were proportional to the fatty acid composition of WCO used as a raw material. Other than the methyl ester components, propanediyl ester which contained an OH functional group was also formed. The formation of this component was due to the molar ratio of methanol and oil used was still less than required, leading to the imperfect glycerol formation and hence separated from the product following the reaction mechanism in Figure 6. This was also proportional to the biodiesel characteristics where it has not met the required Indonesian nor European standard yet.

### 3.10. Catalyst Reusability

The catalyst reusability test was carried out using the highest biodiesel yielding catalyst (KI/HAP 5%) with a simultaneous esterification and transesterification reaction method and three repetitions. Before the reusability test, methanol and oil which stuck to the catalyst were washed with n-hexane, then dried at 110 °C. The yields obtained were 85.6%, 83.8% and 72.1%. The decrease in yield obtained was caused by the pore of catalyst being covered with oil in the reaction. Cakraborty and Roychowdhury [30] explained that the decrease in catalytic activity was caused by the leaching of active species in the catalyst to the reaction media when it was used repeatedly. This statement was also supported by Chen et al. [12] where they conducted a leaching study on HAP supported a K_2_CO_3_ catalyst for biodiesel production. The results from the EDS analysis performed between fresh and an eight-cycle-old HAP/K_2_CO_3_ catalyst showed that the doped K content was lower in the eight-cycle-old catalyst, indicating that repeated usage will cause the K compound to leach out, leading to loss of catalytic activity of the catalyst.

**Figure 9:**
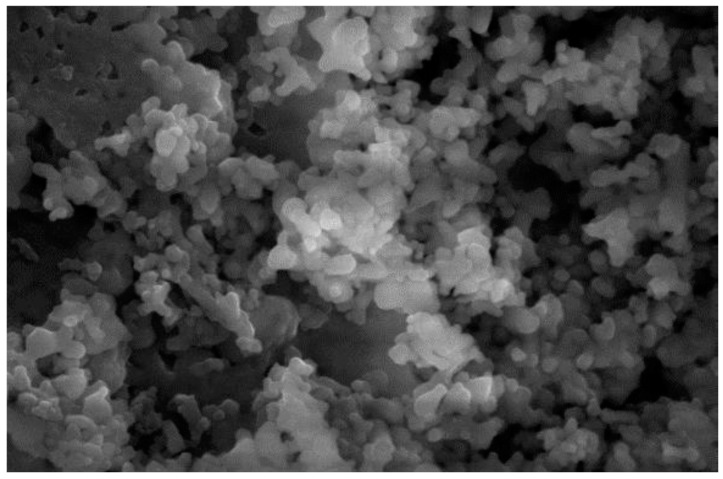
SEM result of the KI/HAP catalyst after three cycles of use.

Figure 9 shows the morphology of the catalyst surface after three cycles of use. The surface of the catalyst is more covered with globular particles when compared to the KI/HAP catalyst in Figure 5a. Those particles which covered the pore could be oil, biodiesel or glycerol. According to Istadi et al. [31], factors that could decrease the catalytic activity of catalyst include species leaching from the catalyst’s active site to the methanol phase [12,31,32], poisoning of the catalyst by the reaction media and also a broken catalyst structure.

Industries generally use homogeneous catalysts such as H_2_SO_4_, HCl, NaOH or KOH to commercially produce biodiesel via a transesterification reaction [33]. Su [34] conducted a recoverability and reusability study on H_2_SO_4_, HCl and HNO_3_ catalysts for biodiesel production from soybean oil. The study concluded HCl has the highest recoverability (99.75%), while HNO_3_ and H_2_SO_4_ recoverability were 69.25 and 57.75% respectively. The reusability of the HCl catalyst was further examined and the results stated after five times of reuse there was no significant difference of FFA conversion (98.19%). Karmee et al. [35] performed a techno-economic evaluation study on biodiesel production using WCO as raw material and H_2_SO_4_, KOH and Novozym-435 as catalysts. It was found that the Novozym-435 catalyst could be recycled 200 times in 30 days without a significant decrease in the biodiesel yield (80%). A HAP supported CaO-CeO_2_ catalyst for biodiesel production with palm oil as a raw material was also investigated for its reusability by Yan et al. [32] where it was found that a catalyst with synthetic HAP had better performance and no significant decrease in yield (83%) after eight times of reuse compared to natural HAP from calcined bones. A similar study on a HAP supported K_2_CO_3_ catalyst conducted by Chen et al. [12] showed above 90% biodiesel yield for 30% *w*/*w* K doped at 600 °C calcination, 85% for 40% *w*/*w* K doped at 600 °C and 79% for 30% *w*/*w* K doped at 500 °C after eight times of reuse. Therefore, it could be concluded that the number of times on which a catalyst can be reused depended on the conversion/yield, as a lower conversion often resulted in higher operating costs and inefficiency of the system. The KI/HAP 5% catalyst synthesized in this study hence could be concluded still favorable after three times of reuse based on the yield obtained (72.100%) compared to the mentioned studies.

## 4. Conclusions

This study explored the possibility of KI/HAP and KIO_3_/HAP catalysts for biodiesel production. The catalyst characterization showed that pure HAP catalyst was successfully synthesized by calcination at 1050 °C for 4 h. After KI/HAP and KIO_3_/HAP impregnations, it showed a crystalline structure shaped in an irregular agglomerate with a surface area of 13.513 m^2^/g and 10.651 m^2^/g, and pore volume 0.0083 cc/g and 0.0065 cc/g respectively. The catalytic activity of KI/HAP and KIO_3_/HAP was evaluated based on yield and % FAME produced. The highest yield was obtained on KI/HAP catalyst for 91.780% and FAME, 59.100%. The results show that the KI/HAP catalyst had better performance and characteristics as compared to the KIO_3_/HAP catalyst as it has a larger surface area, 13.513 m^2^/g and higher yield, 91.780%. The impregnation concentration of KI and KIO_3_ affected the catalyst performance which can be evaluated by the characteristics of the biodiesel produced. Increasing the impregnation concentration of KI and KIO_3_ resulted in a positive response to the yield and % FAME. The sustainability of the catalyst was investigated by its reusability where the results indicated that after three times of use, the catalyst was still favorable and produced 72.100% biodiesel yield. Further research for this work includes a leaching test to confirm the decreased catalytic activity on repeated usage and a biodiesel performance test on deriving energy to further attest the capability of the KI/HAP catalyst in biodiesel production. The optimization of the catalyst under the response surface methodology is also worth studying in future research in this area.

## Figures and Tables

**Table 1:** BET analysis result.

Test Parameter	Unit	Catalyst		
		HAP	KI/HAP (5%) *	KIO_3_/HAP (6%) *
Surface Area	m^2^/g	21.631	13.513	10.651
Pore Volume	cc/g	0.0330	0.0083	0.0065
Pore Radius	Å	16.330	7.380	6.830

Note: * KI/HAP (5%) and KIO_3_/HAP (6%) are the catalysts with the best performance.

**Table 2:** Fatty acid components in WCO.

Component	Composition (% *w*/*w*)	
	This Research	Abidin et al. [24]
Octadecanoic acid (stearic acid)	4.000	4.650
9-Octadecenoic acid (oleic acid)	2.100	33.750
Hexadecenoic acid (palmitic acid)	93.650	13.620
Linoleic acid		43.850
Linolenic acid		4.650
Total	100	100

**Table 3:** Biodiesel characteristic using the HAP/KI catalyst.

Test Parameter	KI impregnation (% *w*/*w*)						Indonesian Standard	European Standard
	1%	2%	3%	4%	5%	6%		
Density (g/cm^3^)	0.873	0.868	0.864	0.861	0.859	0.861	0.850–0.900	0.860–0.900
Viscosity (cSt)	7.803	6.465	6.242	6.688	5.574	5.797	Max. 6 at 40 °C	3.5–5.0 at 40 °C
% FFA	1.876	1.721	1.753	1.817	1.787	1.726	<1	<1

**Table 4:** Biodiesel characteristic using the HAP/KIO_3_ catalyst.

Test Parameter	KI Impregnation (% *w*/*w*)						Indonesian Standard	European Standard
	1%	2%	3%	4%	5%	6%		
Density (g/cm^3^)	0.875	0.871	0.868	0.861	0.863	0.854	0.850–0.900	0.860–0.900
Viscosity (cSt)	8.249	6.242	6.020	6.911	6.020	5.797	Max. 6 at 40 °C	3.5–5.0 at 40 °C
% FFA	1.815	1.879	1.848	1.819	1.789	1.759	<1	<1

**Table 5:** Biodiesel components.

Compounds	Molecular Formulas	Chemical Structure
Hexadecanoic acid, methyl ester (CAS)	C_17_H_34_O_2_	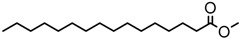
9-Octadecenoic acid, methyl ester (CAS)	C_19_H_36_O_2_	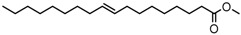
Hexadecanoic acid, 2-hydroxy-1,3-propannediyl ester (CAS)	C_35_H_68_O_5_	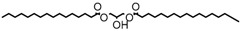
Octadecanoic acid, 2-hydroxy-1,3-propanediyl ester	C_39_H_76_O_5_	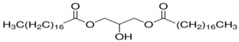
Di-(9-octadecenoyl)-glycerol	C_57_H_102_O_6_